# A High-Quality Assembly and Comparative Analysis of the Mitogenome of *Actinidia macrosperma*

**DOI:** 10.3390/genes15040514

**Published:** 2024-04-19

**Authors:** Jiangmei Gong, Jun Yang, Yan Lai, Tengfei Pan, Wenqin She

**Affiliations:** 1 College of Horticulture, Fujian Agriculture and Forestry University, Fuzhou 350002, China; jiangmeigong@outlook.com (J.G.); laiyan@fafu.edu.cn (Y.L.); tfpan@fafu.edu.cn (T.P.); 2 Laboratory of Ministry of Education for Genetics, Breeding and Multiple Utilization of Crops, Fujian Agriculture and Forestry University, Fuzhou 350002, China; 3 College of Food and Bioengineering, Bengbu University, Bengbu 233030, China; yangj@bbc.edu.cn

**Keywords:** mitogenome, *Actinidia macrosperma*, repetitive elements, codon usage, phylogenetic analysis

## Abstract

The mitochondrial genome (mitogenome) of *Actinidia macrosperma*, a traditional medicinal plant within the *Actinidia* genus, remains relatively understudied. This study aimed to sequence the mitogenome of *A. macrosperma*, determining its assembly, informational content, and developmental expression. The results revealed that the mitogenome of *A. macrosperma* is circular, spanning 752,501 bp with a GC content of 46.16%. It comprises 63 unique genes, including 39 protein-coding genes (PCGs), 23 tRNA genes, and three rRNA genes. Moreover, the mitogenome was found to contain 63 SSRs, predominantly mono-nucleotides, as well as 25 tandem repeats and 650 pairs of dispersed repeats, each with lengths equal to or greater than 60, mainly comprising forward repeats and palindromic repeats. Moreover, 53 homologous fragments were identified between the mitogenome and chloroplast genome (cp-genome), with the longest segment measuring 4296 bp. This study represents the initial report on the mitogenome of the *A. macrosperma*, providing crucial genetic materials for phylogenetic research within the *Actinidia* genus and promoting the exploitation of species genetic resources.

## 1. Introduction

*Actinidia macrosperma*, a naturally wild kiwi, is commonly referred to as “Cat Ginseng” due to its ability to attract cats to exploit it as a natural stimulant and as a remedy for healing wounds [1]. *A. macrosperma* is native to eastern and southern China, mainly found in Zhejiang, Jiangsu, Jiangxi, Guangxi, and Hubei Provinces. The plant material for this study was collected from Desheng Town, Yizhou City, Guangxi Province, on the southern coast of China (108°24′ E, 24°65′ N).

*A. macrosperma* is a medium-sized, deciduous climbing shrub that produces white blooms during spring (April–May) and yields orange fruits in late September. It grows wild on slopes, mountain fronts, moist forest edges, or streams below 800 m above sea level [2]. Along with *A. macrosperma*, there are six other kiwi fruit species rich in beneficial substances such as vitamin C, phenolic compounds, carotenoids, and antioxidant activity (AAC) [3]. Flavonoids in kiwi fruit have health-promoting properties, and certain flavonoids can inhibit the activity of the angiotensin-converting enzyme (ACE), which plays a key role in regulating arterial blood pressure [4].

In China, *A. macrosperma* is also considered a traditional medicinal plant [5], and the roots and stems are widely utilized in treating a spectrum of ailments, including rheumatism, abscesses, joint inflammation, leprosy, jaundice, and abnormal vaginal discharge [6]. Additionally, they have been found to be useful in the management of malignancies, particularly those affecting the digestive system, liver, and lung [7]. However, due to its high medicinal value, excessive excavation has led to a sharp decline in the wild population of this species, and it has even become extinct in some areas. Therefore, it is imperative to closely observe sustainable development and effective protection of *A. macrosperma*. Furthermore, the complete cp-genome of *A. macrosperma* has been sequenced, assembled, and characterized [1].

The mitochondrion is a semi-autonomous organelle in eukaryotic cells, characterized by a small genome known as the mitochondrial genome. It interacts with nuclear and cytoplasmic genetic material to facilitate energy conversion, serving as a biochemical apparatus. Mitochondria synthesize adenosine triphosphate (ATP) through the tricarboxylic acid cycle and oxidative phosphorylation, thereby supplying energy to cells [8]. Additionally, mitochondria play roles in information transmission, cell differentiation, and apoptosis [9]. Research indicates that mitochondria are implicated in cytoplasmic male sterility (CMS), which is maternally inherited [10]. Modern plant breeders are devoted to rearranging mitochondrial genomes to restore plant fertility [11].

The plant mitochondrial genome, despite its complexity and sequencing challenges compared to the chloroplast genome, continues to thrive [12]. By November 2023, the National Center for Biotechnology Information (NCBI) had published 397 chloroplast genomes, 2515 mitochondrial genomes, and 34 plastid genomes (source: https://www.ncbi.nlm.nih.gov/genome/browse#!/organelles/; clicked on 30 November 2023). Although plant mitogenomes typically exhibit a circular genome structure [13], their physical organization still manifested various sub-genomic structures generated by fragments and repeat sequences [14] of linear, circular, and branched structures, along with homologous recombination [15]. Recombination is vital process in DNA replication [16,17,18] for all organisms, even viruses, as it plays a role in the repair and restart of damaged replication forks. Recombination could be classified into two main types, homologous recombination (HR) and non-homologous recombination (NHR), based on its mechanism and the molecules involved [19]. HR serves as the primary route for repairing plant mitochondrial DNA [20], relying on sequences with high similarity for identification and repair. The non-homologous recombination route utilizes limited or non-sequence similarities, potentially resulting in deletions or duplications, particularly in eukaryotes [21,22]. Understanding HR and the factors associated with its regulation contribute to preventing DNA damage and maintaining the stability of the mitochondrial genome. According to the endosymbiosis theory, mitochondria have their origins in an endosymbiotic α-proteobacterium residing within a host cell derived from archaea, eventually evolving into organelles of eukaryotic cells [23]. Therefore, plant mitochondrial HR shares similarities with bacterial HR [24,25,26]. Mitochondrial HR is believed to involve recurrent and interchangeable recombination events with large repeats. The changes that did not affect mitochondrial function were retained, leading to an overall increase in mitochondrial genome size [27]. Plant mitogenomes exhibit significant evolutionary diversity in terms of size, structure, content, intracellular gene transfer (IGT), and interspecific horizontal gene transfer [28]. Nonetheless, the synonymous substitution rates of mitochondrial protein-coding genes display a comparatively greater level of conservation when juxtaposed with those observed in chloroplast and nuclear genomes [29].

Actinidiaceae, composed of three genera, *Actinidia*, *Clematoclethra*, and *Saurauia* [30]. In the past half-century, the Actinidiaceae family has sequenced more than four nuclear genomes (https://www.ncbi.nlm.nih.gov/genome/?term=Actinidia, clicked on 30 November 2023) and over 56 complete chloroplast genomes (https://ngdc.cncb.ac.cn/cgir/genome?input_text=actindiaceae, clicked on 30 November 2023). However, previous reports have only documented a minimal number of complete mitogenomes within this family.

In this study, the assembly and annotation of the mitogenome of *A. macrosperma* were completed, revealing its genomic characteristics and structural features. Repeat sequences were analyzed, and the potential for transfer of chloroplast DNA into the mitogenome was discussed. Additionally, synonymous codon usage (RSCU) was investigated, and the phylogenetic relationships were explored. The results reported in this study offer a distinctive perspective into the mitochondrial evolution of an *Actinidia* species. Moreover, they provide a solid foundation for the effective utilization of available genetic resources and the integration of molecular marker-assisted breeding techniques in the cultivation of *A. macrosperma*.

## 2. Materials and Methods

**Plant materials, genomic DNA extraction, and sequencing.** The *A. macrosperma* materials were originally collected from Desheng Town, Yizhou City, Guangxi Province, China (108°24′ E, 24°65′ N). Genomic DNA was extracted from fresh leaves using the CTAB method [31], followed by quantification using the Qubit fluorescence assay (Invitrogen, Carlsbad, California, USA) and NanoDrop 2000 spectrophotometer (ThermoFischerScientific, Waltham, Massachusetts, USA). DNA degradation and contamination were assessed through agarose gel electrophoresis. The DNA that passed the quality check was fragmented using an ultrasonic water bath. The fragmented DNA was then used to prepare libraries for sequencing. The DNA underwent sequencing using the Nanopore platform (PromethION, Oxford Nanopore Technologies, Oxford, UK) and Illumina HiSeq 2500 platform (Illumina, San Diego, CA, USA). The ONT long-reads generated 20.7 Gb sequencing data. Regarding the NGS short-reads, Illumina PE150 (paired-end 150 bp) sequencing was employed, resulting in 12.69 Gb of raw data, which included 84,576,750 raw reads. Both of the ONT and NGS raw reads were submitted to NCBI (SRR27379599 and SRR27379600).

**Mitogenome assembly and annotation.** The mitochondrial genomes were assembled from ONT reads using SMARTdenovo with its default settings [32]. In order to enhance the precision and efficacy of the mitochondrial genome sequences, the ONT and NGS clean reads underwent refinement through the utilization of minimap2/miniasm [33]. BWA (v0.1.19) [34], SAMtools (v0.1.19) [35], Racon (v1.4.20) [36] and Pilon (v1.23) [37] were utilized for aligning the ONT reads to the assembled mitogenomes. The annotation of the mitochondrial genomes was performed using Geseq (https://chlorobox.mpimp-golm.mpg.de/geseq.html; clicked on 23 October 2023) online with *A. arguta*’s mitogenome (GenBank:MH559343) [38] serving as the reference. Circular maps of the mitochondrial genomes were generated using Ogdraw [39]. Additionally, the assembled sequences of *A. macrosperma* deposited in GenBank under the accession number: OR466481 (https://www.ncbi.nlm.nih.gov/nuccore/OR466481.1/ clicked on 30 November 2023).

**Repeat Sequences.** The analysis of simple sequence repeats (SSRs) was conducted using MISA [40] (https://webblast.ipk-gatersleben.de/misa/, clicked on 30 November 2023) with the parameters set to ‘1-10 2-5 3-4 4-3 5-3 6-3’. Tandemly repeated sequences were identified utilizing the Repeats Finder [41] (v4.09, https://tandem.bu.edu/trf/trf.html, clicked on 30 November 2023) software with default configurations. Dispersed repeats were predicted employing REPuter [42] (https://bibiserv.cebitec.uni-bielefeld.de/reputer, clicked on 30 November 2023) with the following parameters: ‘Hamming Distance 3, Maximum Computed Repeats 5000, Minimal Repeats Size 30′, and ‘e-value cut-off of 1 × 10^−5^’ for filtering criterion.

**Chloroplast-mitochondrion-DNA transfer.** The cp-genome of *A. macrosperma* (MN520000.1) was acquired through the NCBI Organelle Genome Resources Database (https://www.ncbi.nlm.nih.gov/genome/browse#!/organelles/, uploaded on 16 August 2020). Detection of transferred DNA fragments between chloroplast and mitochondrion genome was carried out using BLASTN, employing specified criteria: a matching rate of ≥80%, E-value of ≤1 × 10^−10^, and a minimum length of ≥40 [43]. Visualization of the results was conducted utilizing the software Tbtools [44] (version: 2.041, https://github.com/CJ-Chen/TBtools-II/releases clicked on 30 November 2023), leveraging its advanced circos module.

**Synteny Analyses.** The software MUMmer [45] (version: 3.23, http://mummer.sourceforge.net/ clicked on 30 November 2023) was employed to align the target genome to the reference genome, establishing a broad spectrum of linear relationship between the genome. To confirm the relative positional arrangement of specific regions, BLASTN was employed with the following parameters: a matching rate of ≥85%, E-value of ≤1 × 10^−5^, and a minimum length of ≥100. The creation of a parallel figure was achieved through the utilization of a custom Perl script.

**Phylogenetic Analyses.** Phylogenetic tree construction based on the core genes of mitogenomes involved clustering protein sequences from multiple samples. The cd-hit [46,47] (version 4.6.1, https://www.bioinformatics.org/cd-hit/ clicked on 30 November 2023) was utilized for clustering, considering parameters such as identity and comparison length. The clustering of protein sequences was performed according to the software’s analysis results. Pairwise sequence alignment was conducted using criteria including an identity threshold of ≥0.4 and alignment lengths equal to or greater than * 0.4. Identification of single-copy core genes was followed by protein sequence alignment using software MUSCLE [48] (version: 3.8.31, http://www.drive5.com/muscle). The resulting data were then utilized in constructing evolutionary trees using the NJ method (Neighbor-Joining method) through TreeBeST [49] (version: 1.9.2), with 1000 bootstrap replicates. Visualization of the phylogenetic trees was achieved using the web-based tool iTOL [50] (https://itol.embl.de/ clicked on 30 November 2023).

**Substitution Rate Calculation.** To predict the nonsynonymous substitution rate (Ka) and synonymous substitution rate (Ks) value, the MA algorithm was employed to estimate the Ka/Ks ratios of genes in each reference species compared to *A. macrosperma*. Subsequently, then the Ka/Ks value for the same gene was calculated. Detection of the Ka/Ks ratios for 14 protein-coding sequences obtained from the mitogenomes were detected utilizing KaKs_calculator [51] (V2.0, https://sourceforge.net/projects/kakscalculator2/ clicked on 30 November 2023).

**Codon usage bias analysis.** The CodonW software (version: 1.4.4, https://codonw.sourceforge.net/ clicked on 30 November 2023) was employed to conduct codon bias analysis on the mitogenomes, generating parameters such as the effective number of codon (Nc), GC and GC3, relative synonymous codon usage (RSCU). R with ggplot2 package [52] was employed to create a box plot illustrating the Ka/Ks values and a bar plot representing the RSCU values.

## 3. Results

### 3.1. The Mitogenome Characteristics of Actinidia macrosperma

**Figure 1:**
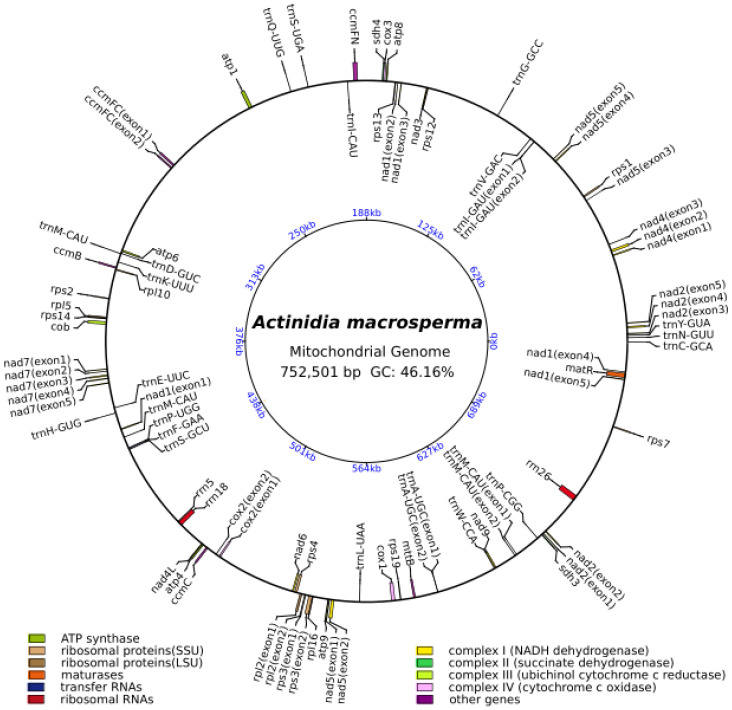
The circular representation of the *A. macrosperma* mitogenome depicting its genomic characteristics, with the genes transcribed in a clockwise direction depicted inside the circle, while those transcribed counterclockwise were illustrated on the outside. The color scheme was assigned based on the functional classification of the genes. The innermost blue numbers represent the mitogenome scale.

The mitogenome of *A. macrosperma* was reconstructed as a circular molecule comprising 752,501 base pairs (bp) (Figure 1). The annotations revealed a total of 39 protein-coding genes (PCGs), 23 tRNAs and three rRNAs in the *A. macrosperma* mitogenome (Tables S1 and S2). The PCGs of *A. macrosperma* comprise five ATP synthase genes, 10 small subunits of ribosome proteins’ genes (SSU), four large subunits of ribosome proteins’ genes (LSU), a maturase, nine NADH dehydrogenase genes (complex I), two succinate dehydrogenase genes (complex II), four cytochrome *c* biogenesis genes (complex III), three cytochrome *c* oxidase genes (complex IV), a transport membrane protein gene, and an Apocytochrome *b*.

### 3.2. Repeat Analysis

**Figure 2:**
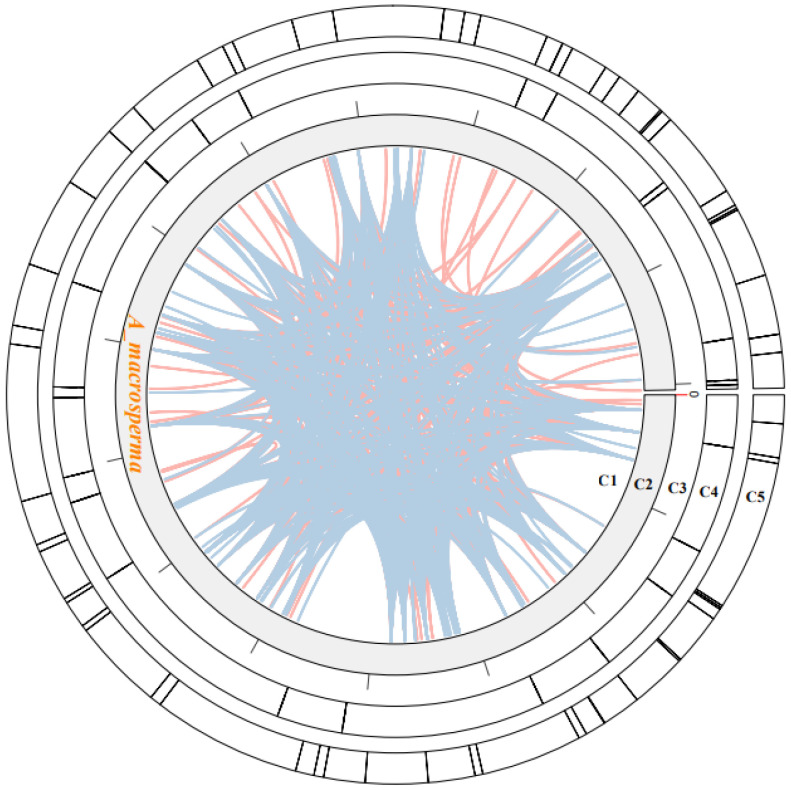
The examination of repeats within the *A. macrosperma* mitogenomes was depicted as follows: Circle1 (C1) illustrated the dispersed repeats, where the connected blue arcs denoted forward repeats, while the pink arcs represented palindromic repeats. C2 showcased the *A. macrosperma* mitogenomes, with a scale of 50 kb on C3. Tandem repeats were visualized as short bars in C4. Microsatellite sequences detected by MISA were delineated in C5.

Within plant mitogenomes, a notable characteristic lies in the abundant occurrence of repetitive sequences, varying in size and dimension. These repetitions are classified into three categories: small (<50 bp), intermediate (50–500 bp), and large (>500 bp) [53]. Additionally, tandem repeats of DNA, referred to as SSRs or microsatellites, consist of 1 to 6 bp units [54]. Approximately 63 SSRs were identified within the *A. macrosperma* mitogenomes (Figure 2; Table S3). Predominantly, the SSRs featured a singular nucleotide repeated unit, notably A/T, constituting 55.56% of all identified SSR repeats. Nevertheless, these SSRs were evenly distributed throughout the surveyed mitogenomes. A. macrosperma harbored 35 mono-, 13 di-, 2 tri-, 11 penta-, and two hexa-repeat units. Furthermore, 25 tandem repeats were identified in the A. macrosperma mitogenomes (Figure 2; Table S4). Further investigation may explore the potential utility of these repetitive sequences in DNA fingerprinting, particularly for applications in molecular marker-assisted breeding.

Dispersed repeats significantly contributed to the augmentation of genetic diversity, exerting a crucial influence on genome evolution [55]. Four types of dispersed repeats were identified: forward, reverse direction, complementary, and palindromic repeats [56]. Within the *A. macrosperma* mitogenome, forward repeats constituted 49.1% of the total repeats, while palindromic repeats accounted for 50.9%. The most extended fragments were 337 bp forward repeats from *A. macrosperma* (Figure 2; Table S5).

### 3.3. Sequence Similarity between Mitogenome and Cp-Genome

**Figure 3:**
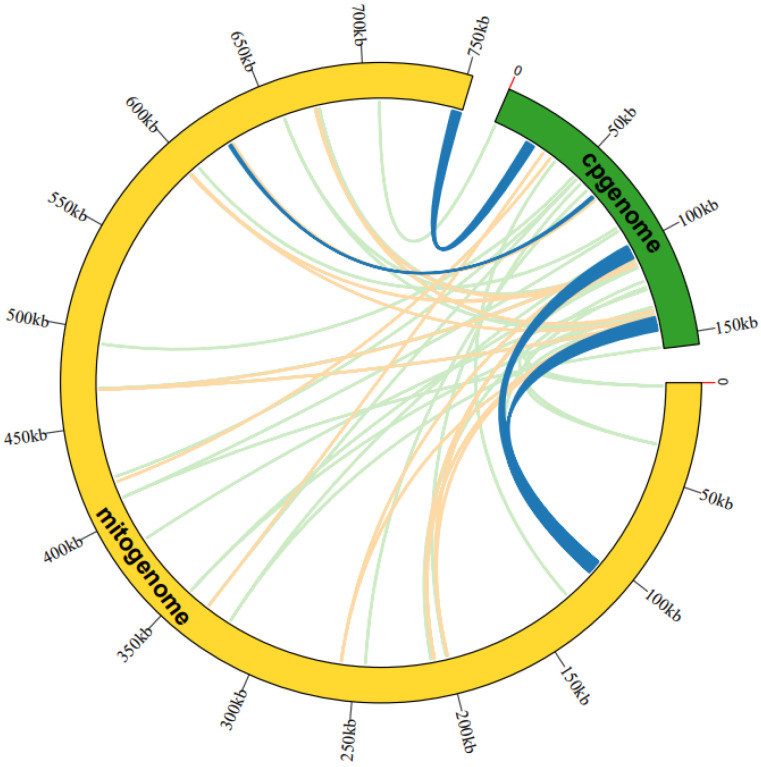
An examination was undertaken regarding the transfer of mitogenome sequences from *A. macrosperma* cp-genomes. The outer arcs, represented in yellow and green, corresponded to the mitogenome and cp-genome, respectively, while the inner arcs depicted homologous DNA fragments. Fragments with alignment lengths exceeding 1000 bp were denoted in dark blue, those below 100 bp were depicted in jade-green, and fragments falling within this range were shown in orange. The outer arcs exhibited a scale at 50 kb intervals.

The analysis of sequence similarity indicated that the 17,783 bp sequences discovered in the *A. macrosperma* mitogenome likely originated from the corresponding cp-genome (Figure 3; Table S6), constituting 2.36% of the mitogenome’s sequence. A total of fifty-three homologous fragments were identified, with the longest measuring 4296 bp, between the mitogenome and cp-genome. Furthermore, these sequences’ homologous fragments encompassed eight chloroplast genes (rpoC1, ndhB, rps7, rps12, rrn16, psbF, psbE, petL) from *A. macrosperma* cp-genome and four mitochondrial genes (trnA-UGC, trnI-AGU, trnV-GAC, trnW-CCA) from *A. macrosperma* mitogenome.

### 3.4. Collinearity Analyses of Mitogenome in Actinidia

The mitogenome sequences from six Actinidia species were retrieved from NCBI’s available mitogenome resources (Table S7). These genomes demonstrate comparable GC contents, ranging between 42.0% and 46.2%. However, there exists a notable diversity in genome sizes, spanning from 768,883 to 1,020,276 bp. Upon re-annotation of these mitogenomes, it was noted that certain core protein-coding genes (PCGs) were present in multiple copies, with four out of seven mitogenomes displaying re-annotated multiple-copy PCGs.

**Figure 4:**
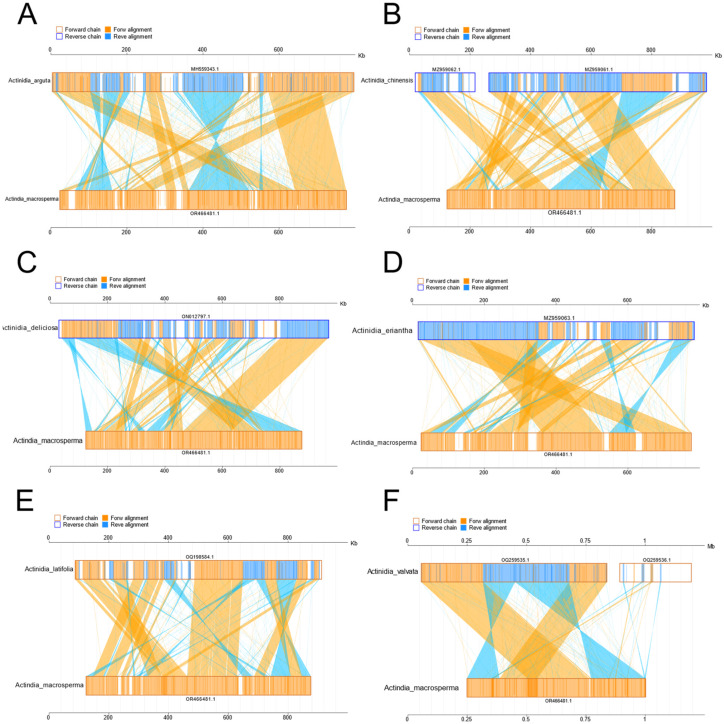
The diagrams delineate collinear regions observed among disparate mitogenomes in additional *Actinidia* species when compared to *A*. *macrosperma* ((**A**): *Actinidia arguta*; (**B**): *Actinidia chinensis*; (**C**): *Actinidia deliciosas*; (**D**): *Actinidia eriantha*; (**E**): *Actinidia latifolia*; (**F**): *Actinidia valvata*). The outer frame colors represent the orientation of the mitogenomes sequences (orange indicating the forward direction and blue indicating the reverse direction), while the fill colors represent the alignment status (orange indicating forward alignment and blue indicating reverse alignment).

The investigation into collinearity among *Actinidia* mitogenomes aimed to assess genome rearrangements across different lineages. Using *A. macrosperma* mitogenomes as reference sequences, collinearity analyses revealed varying lengths of syntenic stretches across sampled species, ranging from less than 30 kb between *A. arguta* to more than 50 kb between *A. valvata* (Figure 4). These findings imply that significant rearrangements occur in mitochondrial genomes of *Actinidia* species with their divergence, leading to alterations in mitochondrial genomic synteny.

### 3.5. Phylogenetic Analysis

**Figure 5:**
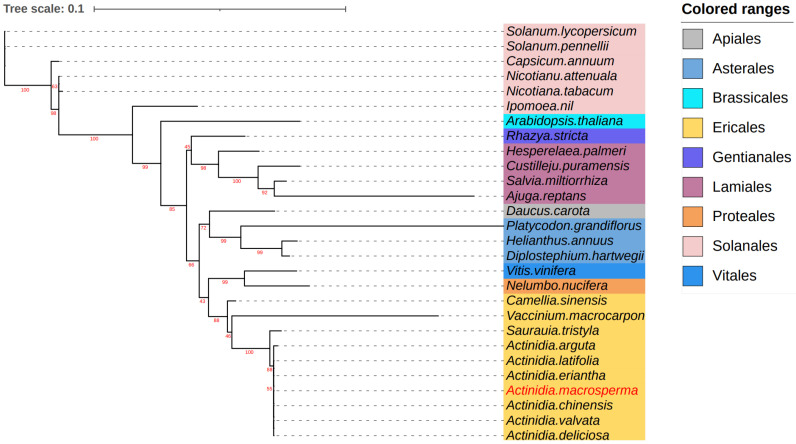
The analysis delved into the phylogenetic relationships involving *A. macrosperma* and other species. Utilizing twelve core genes from the mitogenomes, a phylogenetic tree was constructed based on protein sequences. The mitogenomes under study were highlighted in a red font, while variously colored backgrounds were employed to denote the taxonomic classification of orders within the investigated species. The bootstrap values were denoted by the red numbers positioned on the branches of the phylogenetic tree.

To explore the evolutionary dynamics of the *A. macrosperma* mitogenome, phylogenetic analyses were performed on the mitogenomes of *A. latifolia*, *A. valvata*, and 28 other related species. A set of 12 core genes (*atp8*, *matR*, *ccmB*, *ccmFN*, *nad9*, *nad3*, *atp1*, *atp4*, *rps12*, *ccmC*, *nad6*, *cox3*) was used for phylogenetic analysis. The trees generated from the core genes of mitogenomes revealed that *A. macrosperma* and *A. chinensis*, along with *A. valvata* and *A. deliciosa*, formed a cluster (Figure 5).

### 3.6. Substitution Rates

**Figure 6:**
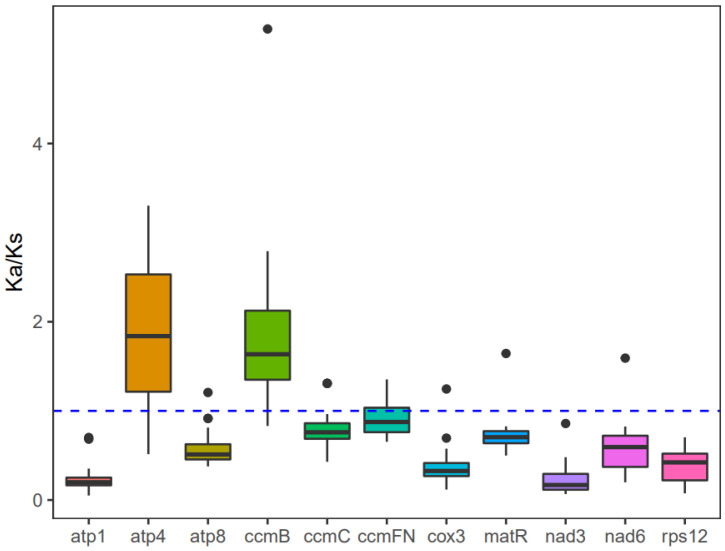
The Ka/Ks values for the individual mitochondrial genes in seven species of *Actinidia* are illustrated in the box diagram. The *x*-axis and *y*-axis represent protein-coding genes and Ka/Ks values, respectively.

The investigation into the evolutionary rate of mitochondrial genes in *A. macrosperma*, Ka/Ks values were detected for 12 core protein-coding genes (Table S8). Consequently, positive selection was inferred for *atp4* and *ccmB* due to their Ka/Ks ratios surpassing 1, while genes with lower Ka/Ks ratios were likely under purifying selection (Figure 6). Particularly, the *atp1* gene displayed minimal variation and a low Ka/Ks ratio, underscoring its high conservation and pivotal role in mitogenome functionality.

### 3.7. Codon Usage Bias Analysis

**Figure 7:**
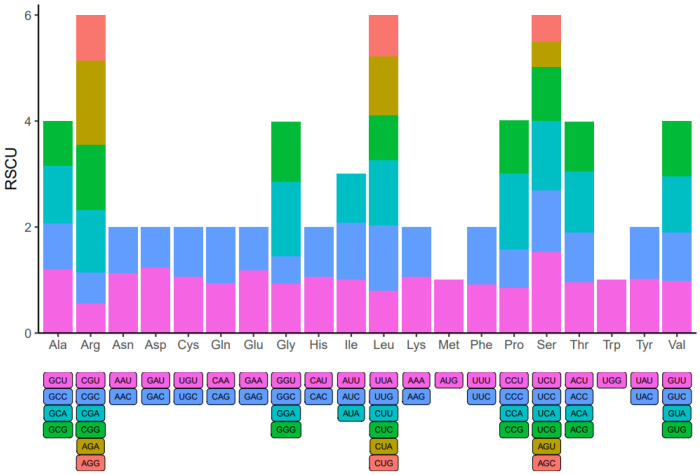
Codon usage bias was observed in the mitochondrial PCGs of *A. macrosperma*. The term RSCU denotes relative synonymous codon usage.

Within the genome of eukaryotic organisms, 20 different amino acids are encoded by 64 codons, with multiple codons coding for each amino acid except for Methionine and Tryptophan. Codon usage varies significantly across species due to codon degeneracy. An examination of codon usage was conducted on all mitochondrial protein-coding genes (PCGs) in *A. macrosperma* (Figure 7 and Table S9). Codons with a relative synonymous codon usage (RSCU) exceeding 1 were preferred by amino acids, suggesting a universal preference for codon usage among mitochondrial PCGs. For instance, Arginine (Arg) prefers AGA codons, with the maximum RSCU value among *A. macrosperma* mitochondrial PCGs at 1.60. Serine (Ser) closely follows, showing a preference for UCU (RSCU = 1.53). Additionally, CCA (Pro), GGA (Gly) and UCA (Ser) emerge as the three most common codons in *A. macrosperma,* potentially reflecting a preference shaped by long-term evolutionary selection. Moreover, in comparison to six other *Actinidia* species (Table S9), the result indicates that the RSCU values of *A. macrosperma*’s closely resemble those of *A. delicese*, while significant differences exist in the RSCU values of the other five species.

## 4. Discussion

The present investigation achieved the successful assembly of high-quality mitogenomes for *A. macrosperma* by integrating sequence datasets obtained from Illumina short-reads and Oxford Nanopore long-reads. Sequence assembly elucidated that the mitogenome of *A. macrosperma* is represented by a single, circular molecule spanning 752,501 base pairs, displaying a GC content of 46.16% (Figure 1). Notably, the mitogenomes of *A. macrosperma* and the other six *Actinidia* species had 39 PCGs each, whether they contained one or two molecules, indicating a high conservation of protein-coding gene numbers in Actinidiaceae family mitogenomes [57,58,59]. In addition, 15 introns were found in the mitogenome of *A. macrosperma*, compared to the presence of 13–15 introns in the other six *Actinidia* species (Table S7). This evidence underscores the relative stability of mitochondrial intron content across the majority of land plant lineages, despite occasional acquisitions and numerous convergent losses over evolutionary time [60].

Repetitive elements, characterized by the presence of similar or symmetrical fragments, manifest at various loci within the genome. This phenomenon extends to both intra-species genomic regions and inter-species genomic comparisons. Studies have revealed the prevalent distribution of repeats throughout plant mitogenomes, with these sequences exhibiting limited conservation across species and a predominance of short repeats [61]. Among these, the majority of simple sequence repeats (SSRs) consist of single-nucleotide (A/T) repeats, constituting approximately 55.56% of all SSRs, likely influenced by the low GC content typical of mitochondrial genomes. This trend parallels observations in the mitogenome of *A*. *latifolia* [59]. Moreover, a significant abundance of dispersed repeats, comprising primarily forward and palindromic repeats in roughly equal proportions, was identified within the mitogenome. Similar findings have been reported in the mitochondrial genomes of *Actinidia* [59] and other species, such as *Gleditsia sinensi* [62].

Due to the unique genomic structure and evolutionary dynamics, the plant mitogenome displays heightened susceptibility to the integration of exogenous DNA [27]. Concurrently, exogenous DNA is prevalent within plant mitochondria [63]. Several investigations have highlighted notable resemblance between the mitogenome and chloroplast genome, suggesting occurrences of DNA transfer events [64,65]. These substantial homologous segments are believed to have played a pivotal role in the extensive evolutionary processes of eukaryotes, fostering genetic diversity. Additionally, studies have unveiled a bifurcated origin of tRNA genes in plant mitochondria, with a portion inherited from mitochondrial ancestors and another acquired via horizontal gene transfer (HGT) from chloroplasts [66]. The identification of chloroplast-derived tRNA genes in the *A. macrosperma* mitogenome has been accomplished. Remarkably, four mitogenome tRNAs, specifically trnA-UGC, trnI-GAU, trnV-GAC, and trnW-CCA, exhibited significant similarity to the entire sequence of the chloroplast genome, collectively representing 17.4 percent of all tRNAs (Table S6). Furthermore, trnW-CCA is widely distributed across angiosperm mitogenomes, exhibiting homology with chloroplasts [67].

Assessing the level of collinearity between various species can provide insights into their evolutionary divergence, with genetic relationships often inferred from collinear patterns. Collinearity analysis was undertaken to investigate mitochondrial genome DNA rearrangements across different kiwi fruit species (Figure 4). Line diagrams showed that *A. macrosperma* were closely related to *A. valvata*, with the largest collinearity region about 50 kb, while A. *arguta* has the smallest collinearity region, about 30 kb. The collinearity of distantly related species was relatively small, potentially resulting from significant DNA rearrangements occurring in successive generations in the past [68]. Meanwhile, evolutionary analysis based on 12 core genes also supports this conclusion in which the relationship of *A. macrosperma* was closer to *A. valvata*, *A. chinensis*, and *A. deliciosa*, while it was distantly related to *A. arguta* of Kiwifruit species (Figure 5). Earlier research findings was consistent with the primary conclusions mentioned above, as indicated by evolutionary studies based on the *Actinidia* mitogenome, showing a closer genetic distance among *A. chinensis*, *A. deliciosa*, and *A. valvata*, while being more distantly related to *A. valvata* [59].

Examining the synonymous and nonsynonymous substitution rates offers valuable understanding regarding the impact and extent of natural selection on protein evolution [69]. Through examination of the correlation between SNPs and gene mutations, comprehensive insights into genome-wide gene mutations can be obtained, enabling the inference of the evolutionary trajectory of the entire species and its underlying causes: positive selection for active adaptation to the environment, negative selection resulting from environmental pressures, or neutral selection to maintain a balance between the two. Consistent with previous research [52,59,70], the majority of mitochondrial genes underwent neutral evolution under the influence of negative selection, displaying a high degree of conservation. However, positive selection may impact genes such as *atp4* and *ccmB*, as indicated by their dN/dS ratio surpassing 1. The protein encoded by *atp4*, a component of the F1F0-ATPase subunits, contributes to the conversion of proton flow into ATP within the matrix, serving as a vital energy source for cellular activities [71].The *ccm* gene family encoded by the *ccmB* gene is important for the biosynthesis of cytochrome c, which is derived from early prokaryotic cells from the plant mitotic genome [72]. Previously, it had been reported that the ccmB gene is positively selected in *A. valvata* [59] and *Scutellaria baicalensis* [73], and the gene of *atp4* also exhibited highest Ka/Ks values above one in *Diospyros oleifera* [52]. However, the biological process for these observations is still to be explained.

## 5. Conclusions

The mitogenome assembly and annotation of *Actinidia macrosperma*, a Chinese traditional medicine plant belonging to the Actinidiaceae family, were presented for the first time. The comparative analysis encompassing gene structure, repeat regions, homologous fragments with cp-genome, and Ka/Ks codon usage played a crucial role in investigating the characteristics of the *A. macrosperma* mitogenome. These analyses yielded essential insights into the evolutionary history, functional characteristics, and adaptability of the *A. macrosperma* mitogenome, facilitating a better understanding of its distinct features. Comparative analysis, further enhanced our comprehension of the similarities and disparities between the *A. macrosperma* mitogenome and other related species, thereby offering deeper insights into its evolutionary role and mechanisms of ecological adaptation. In this study, the mitochondrial genome of *A. macrosperma* was thoroughly explored from the perspectives of genomic functional structure and genetic evolution, thus contributing valuable genetic resources for phylogenetic investigations and laying the foundation for understanding the evolutionary relationships within the Actinidiaceae family.

## Data Availability

The accession number generated for this study is available on NCBI. The raw sequencing data have been deposited in NCBI (https://www.ncbi.nlm.nih.gov/, Clicked on 23 December 2023) with accession numbers: PRJNA1056159, SAMN39090875, SRR27379599 and SRR27379600 (https://www.ncbi.nlm.nih.gov/sra?LinkName=biosample_sra&from_uid=39090875). And the assembled sequences accession number of *A. macrosperma* mitogenome is OR466481 (https://www.ncbi.nlm.nih.gov/nuccore/OR466481.1/).

## Supplementary Materials

The following supporting information can be downloaded at: https://www.mdpi.com/article/10.3390/genes15040514/s1, Table S1. Gene composition in the mitogenome of *Actinidia macrosperma*. Table S2. Gene information in the mitogenome of *Actinidia macrosperma*. Table S3. Microsatellite repeats in the *Actinidia macrosperma* mitogenome. Table S4. Tandem repeats in the *Actinidia macrosperma* mitogenome. Table S5. Dispersed repeats in the *Actinidia macrosperma* mitogenome. Table S6. Length of transferred DNA from *Actinidia macrosperma* cp-genome. Table S7. Features of seven mitogenomes belonging to the *Actinidia*. Table S8. The Ka and Ks for 12 core PCGs with Reference to *Actinidia macrosperma*. Table S9. The RSCU values with 39 PCGs of the *Actinidia*.
